# 

**Published:** 1911-04-22

**Authors:** 


### MEDICAL APPOINTMENTS, ETC.

Mr. R. H. Foa has been elected a Vice-President of the Gravesend Hospital.

At the monthly meeting of the Board of Management of the Bradford Royal Infirmary, held on Friday, April 7, Dr. A. Bronner was appointed honorary consulting laryngologist, and the best thanks of the Board were accorded to him for his services as honorary laryngologist since 1891.

Mrs. Fanny Maria Kemp, of Pangbourne, has bequeathed £2,000 to Westminster Hospital to endow a bed to be known as the "Sir Benjamin Baker bed, given by his sister, Mrs. Fanny Maria Kemp, in memory of him," and £1,000 to the Royal Berkshire Hospital, Reading.

Mr. Robert Halford, of Nottingham, has left £1,000 to the Nottingham Children's Hospital, to endow a cot to be called the "Carrie Halford" Cot, in memory of his late wife; and £100 to Nottingham General Hospital and to the Nottingham General Dispensary.

Mr. Simon Symons, stockjobber, has left £1,000 to the Norwood Cottage Hospital, conditional upon the bed known as the "Florence Bed," hitherto supported by him, being henceforward known as the "Symons Bed," and failing the acceptance of this bequest on these conditions, this sum is to revert to King Edward's Hospital Fund for London.

Sir Charles Rackham Gilman, of Eaton, Norwich, has left £300 to the Norfolk and Norwich Hospital; £200 to the Jenny Lind Infirmary, Norwich; and £100 each to the Norwich Asylum and School for the Indigent Blind, the Norfolk and Norwich Eye Infirmary, the Children's Convalescent Home, Great Yarmouth, and to the Lowestoft Convalescent Home.

### Gifts and Bequests.

The Samaritan Free Hospital for Women, Marylebone Road, has received £50 from the Clothworkers' Company.

Among the latest contributions for King Edward's Hospital Fund is an annual subscription of £500 from Messrs. N. M. Rothschild and Sons.

Mr. George Walter Tait, M.R.C.S., of The Laurels, Knowle, Warwickshire, who died on March 27, aged sixty-five, left estate valued at £32,173 gross. The testator left £1,000 to the United Kingdom Beneficent Association, to found an annuity or annuities in memory of his sister, Susan Harriette Tait, and to bear her name, and to be for spinster daughters of deceased medical men or Church of England clergy; £500 to the Royal Medical Benevolent College; and £100 each to the Midland Counties Home for Incurables, Leamington, and the Midland Counties Asylum, Knowle.

The Hon. Henry Lorton Bourke, of County Meath, Derbyshire, and Berkeley Square, stockbroker, who left £521,797, has left the balance of the income of the £200,000 to be used for the providing of support of the Bourke clubs, to provide "Bourke grants" for any or all of the following purposes in the discretion of the trustees—namely, The Navan Infirmary, so long as it is not conducted to the prejudice of Protestant inmates, in which case no grant is to be made; the National Hospital for Consumption for Ireland; the Adelaide Hospital, Dublin; the London Hospital, the Charing Cross Hospital, the Middlesex Hospital, St. George's Hospital, the Cancer Hospital.

### BUILDINGS CONTEMPLATED AND IN PROGRESS.

Bradford.—The city architect, Mr. W. Williamson, has submitted a scheme to the Corporation for the erection of a sanatorium at Grassington; it provides fifty beds at an estimated cost of £16,500; an alternative scheme for sixty-six beds at a cost of £17,680 is also under consideration. The buildings will be at an altitude of 800 feet.

Clonmel.—Mr. J. F. Fuller, architect, Dublin, is in charge of the extension works for improved nurses' accommodation, etc., at the Clonmel Lunatic Asylum. Estimates are now invited, and the drawings, etc., may be seen at Mr. Fuller's office.

Carnarvon.—Mr. Rowland Lloyd-Jones, county architect, has been commissioned to prepare plans for a new hospital in connection with the Carnarvon Workhouse to submit to the Local Government Board.

London.—A hospital for the treatment of mental diseases is projected on Denmark Hill, in proximity to the new King's College Hospital; Dr. Henry Maudsley has offered £30,000 towards the cost of a building estimated to cost inclusive of land between £50,000 and £60,000.

### COMING EVENTS OF THE WEEK.

[Communications for this column must be sent to 29 Southampton Street, Strand, before noon on Wednesday.]

Tuesday, April 25.—Central London Throat Hospital, Gray's Inn Road, W.C.: Lecture on "Accessory Sinuses," by Mr. C. Nourse, 3.45 p.m.

Wednesday, April 26.—Annual General Meeting, Royal Eye Hospital, St. George's Circus, Southwark, 4 p.m.
Annual Meeting Children's Fresh-Air Mission, Court Room, Holborn Municipal Offices, Mayor of Holborn presiding, 3.30 p.m.

Thursday, April 27.—Cancer Hospital, Fulham Road, S.W.: Duke of Connaught opens new Cancer Research Institute, 3 p.m.

Friday, April 28.—Central London Throat Hospital, Gray's Inn Road, W.C.: Special Clinical Demonstration by Dr. Abercrombie.

### APPOINTMENTS VACANT.

Full particulars of these vacancies can be obtained on reference to our advertisement columns:

Cambridge County Asylum.—Second Assistant Medical Officer.

Hospital for Sick Children, Great Ormond Street, W.C.—Assistant Casualty Medical Officer.

Leeds General Infirmary.—Ophthalmic Officer.

Leeds General Infirmary (Ida and Robert Arthington Hospital.—Medical Officer, Obstetric Officer, Two House Physicians, One House Surgeon, One Ophthalmic House Surgeon.

Royal Albert Hospital, Devonport.—Assistant Resident Medical Officers.

Royal United Hospital, Bath.—House Surgeon.

